# Editorial: Cutting-edge systems and materials for brain-inspired computing, adaptive bio-interfacing and smart sensing: implications for neuromorphic computing and biointegrated frameworks

**DOI:** 10.3389/fnins.2023.1321387

**Published:** 2023-10-26

**Authors:** Guobin Zhang, Teng Ma, Bo Wang, Desmond K. Loke, Yishu Zhang

**Affiliations:** ^1^School of Micro-Nano Electronics, Zhejiang University, Hangzhou, Zhejiang, China; ^2^ZJU-Hangzhou Global Scientific and Technological Innovation Center, Hangzhou, Zhejiang, China; ^3^Department of Applied Physics, The Hong Kong Polytechnic University, Kowloon, Hong Kong SAR, China; ^4^Department of Science, Mathematics and Technology, Singapore University of Technology and Design, Singapore, Singapore

**Keywords:** neuromorphic computing, brain-inspired computing, adaptive bio-interfacing, smart sensing, bio-integrated frameworks

## Introduction

Nowadays, humanity is entering the age of artificial intelligence (AI). The development of the AI era requires more and more data, algorithms and computing power. However, the mode of increasing computing power by increasing the transistor integration of integrated circuits is no longer sustainable since Moore's law and Dennard scaling have both approached the bottleneck. In response to these problems with traditional CMOS architectures, brain-inspired computing, adaptive bio-interfacing, and smart sensing, inspired by the architecture and workings of the biological brain, are the way forward. At present, brain-inspired computing, adaptive bio-interfacing, and smart sensing as a whole are still in the stage of single-point technological breakthroughs and facing many challenges. Apparently, cutting-edge systems and materials are sources of dealing with the issues mentioned above.

In this Research Topic, we will present applications of cutting-edge systems and materials in three major areas: brain-inspired computing, adaptive bio-interfacing, and smart sensing. At the same time, the research theme will also seek to discuss the implications of cutting-edge systems and materials for the development of neuromorphic computing as well as bio-integrated frameworks outside of their applications in these cutting-edge broad areas.

## Cutting-edge systems and materials for brain-inspired computing

In this section, we first review the research and applications of cutting-edge systems and materials in the field of brain-inspired computing recently.

Spiking neural networks (SNNs) are more competitive with non-spiking models in many computer vision tasks. Using a brain-inspired neural circuit evolution strategy and a rich set of neural circuit types, Shen et al. (2023) evolved SNNs that greatly enhance perceptual and reinforcement learning tasks while combining online and offline deep reinforcement learning algorithms. Yu M. et al. proposed a novel optimization algorithm for TTFS-based SNN systems that improved the accuracy of SNNs in terms of TTFS encoding and training. Yu C. et al. proposed a spatiotemporal synaptic connection SNN model by combining temporal convolution and attention mechanisms to enhance the spatiotemporal receptive field of synaptic connections.

Nevertheless, the SNN training algorithms are not mature enough for brain-inspired computing. Thus, realizing the conversion between SNN and ANN helps to improve the accuracy of brain-inspired computing. Bu et al. (2023) achieved high-accuracy and high-performance SNN-ANN conversion with ultra-low latency (4 time-steps) and zero conversion error. A clip-floor-shift activation function replacing the ReLU activation function in the ANNs was proposed to better approximate the activation function of SNNs.

In addition to research on cutting-edge neural networks such as SNNs in the field of brain-inspired computing, there is some very interesting cutting-edge research that can provide guidance on the direction of brain-inspired computing. Li et al. explained the scale-free properties the relationship between energy consumption and synchronization of the neural network structure of the brain from the perspective of energy efficiency based on the Hodgkin-Huxley model. Wang et al. presented a dual-function state neural network that enabled sustained neural firing and activity-silent working memory. All of the above work can provide more possibilities to further utilize different neural activities in the brain to simulate complex brain-inspired computing functions.

As the size of devices based on traditional bulk materials continues to minimize, they face problems such as increased short-channel effects and lower threshold voltages, which in turn lead to increased energy dissipation. In order to break through the existing limitations of conventional bulk materials, Zhao et al. reported a dynamic LiNbO_3_-based memristor for reservoir computing. Based on current trends, research interest begins to move to low-dimensional materials in the field of neuromorphic devices due to their extreme flat surface, feasibility of hetero-integration and unique electronic properties. Kamaei et al. (2023) integrated Si:HfO_2_ ferroelectric gates with WSe_2_/SnSe_2_ 2D heterojunctions to construct a reconfigurable device. Sun et al. (2023) reported an ionic 2D CuInP_2_S_6_ (CIPS)-based amnesia that could realize up to 1,350 linear conductance states by controlling the migration of internal Cu ions in the CIPS.

In the future, we can further investigate the efficient deployment of neural network learning training algorithms in combination with advanced materials (e.g., 2D materials, etc.) used for brain-inspired computing.

## Cutting-edge systems and materials for adaptive bio-interfacing

In this section, we review the applications of cutting-edge systems and materials in the field of adaptive bio-interfacing.

To date, cutting-edge systems for adaptive bio-interfacing have been converted to relatively mature applications in many fields, such as medicine. Full of practical meaning, for patients with spinal injuries, Lorach et al. (2023) utilized a digital bridge between the brain and spinal cord to restore communication between the brain and the areas of the spinal cord that produce walking functions.

Because achieving robust tissue adaptation and bio-interfacing for bioelectronic devices is one of the key issues to be addressed, research on new materials with excellent electrical, chemical, and mechanical properties is a top priority. Park et al. (2023) presented an intrinsically non-swelling multifunctional hydrogel with excellent comprehensive mechanical properties and rapid self-healing ability, and were able to achieve high electrical conductivity and tissue adhesion of the hydrogel to the target tissues after simple chemical modification. Choi et al. (2023) reported a strain-adaptive adhesive bioelectronic patch that spontaneously adheres to the epicardium within 0.5 s without external stimulation, thus enabling long-term accurate monitoring of ECG signals.

At this stage, cutting-edge research on adaptive bio-interfacing focuses on the biocompatibility of new material aspects, but the development of new systems is understudied. In order to further improve the efficiency and accuracy of bio-signal transmission between the signal source and the bioelectronic device, in-depth research on cutting-edge systems is essential.

## Cutting-edge systems and materials for smart sensing

Here, we finally review of the current state of research on cutting-edge systems and materials for smart sensing.

As a representation of smart sensing, the cutting-edge systems of all-in-one array draw extensive attention from scholars. Zha et al. (2023) demonstrated a fully memristive in-sensor reservoir computing system that can simultaneously sense, decode, and learn from fiber-optic transmission based on its rich dynamic properties, paving the way for future intelligent signal processing systems at the edge. Similarly, Chen et al. (2023) implemented a single bio-inspired visual sensor to efficiently respond to and encode light stimuli in the temporal domain by modeling hierarchical neurons in the insect visual system.

Similar to brain-inspired computing, cutting-edge materials for smart sensing are also dominant by 2D materials. Ci et al. (2023) proposed a two-terminal integrated visual smart sensor device consisting of α-In_2_Se_3_/SnSe ferroelectric p-n heterojunctions, which successfully mimicked basic synaptic function, light adaptation, and associative learning, combined with ferroelectrode polarization inversion to simulate the property that the artificial retina can adapt to bright light stimuli by modulating synapses. Besides 2D materials suitable for the roadmap of “Beyond CMOS,” Hong et al. (2023) reported a copper single-atom catalyst (CuSAC6N6) and constructed a smart biosensing system for glucose in vitro with smart switching of sensitivity and linear detection range based on the adaptive ability of CuSAC6N6.

Both smart sensors and all-in-one sense-memory-computing neuromorphic arrays are currently at a high speed in the development of cutting-edge systems and materials, which is largely driving the development of the two large fields of neuromorphic computing and bio-integrated frameworks in the era of the IoT.

## Conclusion

The success of the current phase of the AI field has led to a boom in learning and mimicking biological sensing and processing systems to build the interactive intelligence systems of the future. Brain-inspired computing can potentially achieve lower power consumption, higher density integration, and enhanced computing efficiency, etc. through the integration of cutting-edge systems and materials. For bio-interfacing, human intelligence and machine intelligence can be linked and integrated to work together through powerful brain-machine adaptive bio-interfacing. Concurrently, the continuous exploration and development of new materials have led to the emergence of a variety of smart sensing materials and systems capable of detecting humidity, temperature, and photoelectrical signals. As research in these three areas continues to progress, the development of neuromorphic computing and bio-integrated frameworks is expected to transition from basic research to a high-speed trajectory in practical applications.

We believe that this topic will inspire more scholars in the field of neuromorphic computing and bio-integrated frameworks and raise the awareness of the general public in the hot areas of brain-inspired science.

## Author contributions

GZ: Writing—original draft. TM: Writing—review & editing. BW: Writing—review & editing. DL: Supervision, Writing—review & editing. YZ: Funding acquisition, Supervision, Writing—review & editing.
